# Sex Differences in Energy Metabolism Need to Be Considered with Lifestyle Modifications in Humans

**DOI:** 10.1155/2011/391809

**Published:** 2011-06-06

**Authors:** Betty N. Wu, Anthony J. O'Sullivan

**Affiliations:** ^1^St. George Clinical School, Faculty of Medicine, University of New South Wales, Sydney, NSW 2052, Australia; ^2^Department of Medicine, St. George Hospital, Kogarah, NSW 2217, Australia

## Abstract

Women have a higher proportion of body fat compared to men. However, women consume fewer kilojoules per kilogram lean mass and burn fat more preferentially during exercise compared with men. During gestation, women store even greater amounts of fat that cannot be solely attributed to increased energy intake. These observations suggest that the relationship between kilojoules consumed and kilojoules utilised is different in men and women. The reason for these sex differences in energy metabolism is not known; however, it may relate to sex steroids, differences in insulin resistance, or metabolic effects of other hormones such as leptin. When considering lifestyle modifications, sex differences in energy metabolism should be considered. Moreover, elucidating the regulatory role of hormones in energy homeostasis is important for understanding the pathogenesis of obesity and perhaps in the future may lead to ways to reduce body fat with less energy restriction.

## 1. Introduction

Fat gain is always considered to be a result of long-term positive energy balance, whereby daily energy intake exceeds expenditure. From the onset of puberty to menopause, women maintain a greater percentage body fat mass (FM) than men despite smaller energy intake per kg lean mass [1] and preferential use of fat as a fuel during exercise compared to men [2]. A potential reason for these findings is that the greater FM in women relates to more efficient fat storage during nonexercising periods, especially postprandial periods [3]. During pregnancy, women deposit between 2.4 to 5.9 kg of body fat, even when undernourished [4]. In well-nourished women, the energy cost of gestation is approximately 370 MJ [5]. How this energy requirement is met is not explained purely by an increase in energy consumption, as past studies failed to demonstrate this in the first half of pregnancy [6, 7]. The reproductive years and gestation are characterised by elevated levels of ovarian hormones. Evidence indicates that oestrogens contribute to the gender differences in FM and the gestational changes in body composition [3]. Human and animal studies have explored possible mechanisms of action by these hormones [8, 9]. When considering lifestyle modifications, the sex difference in energy metabolism needs to be considered. 

The first half of this paper focuses on differences between men and women: the gender differences in FM are outlined, aspects of energy metabolism that may account for these differences are discussed, and the key metabolic roles of ovarian hormones are discussed. Against this backdrop, the second half of this paper focuses on body composition and energy balance during pregnancy.

## 2. Gender Differences in Body Composition Throughout Life

**Figure 1:**
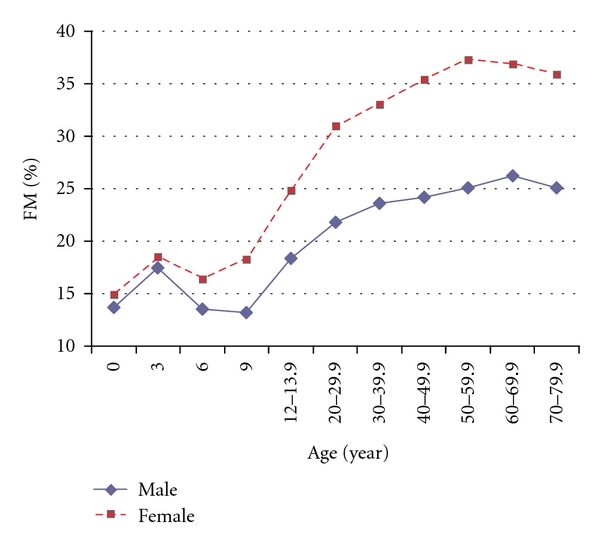
Percentage fat mass (FM) in males and females showing the divergence that occurs at puberty and persists through the pre-menopausal years. Combined data from Chumlea et al. [10] and Fomon et al. [20].

Like many mammals, humans show significant differences in fat-free mass (FFM) and FM between the sexes. The *National Health and Nutrition Examination Survey* III (NHANES III) of 15,912 subjects, showed that non-Hispanic white females aged between 12 and 80 years have a higher percentage of FM than males, starting from puberty and varying from 6% to 11% higher for every decade studied (see Table 1 and Figure 1) [10]. Other studies also support the notion that the significant sexual divergence in body composition commences with puberty [3]. This sex difference holds across all ethnic groups and has been observed in all populations although its magnitude is influenced by ethnic, genetic, and environmental factors [11]. Not only is there a difference in percent FM between the sexes, there is also a well-recognised difference in body fat distribution.

## 3. Gender Difference in Energy Metabolism

It may be postulated that women store more fat because they consume more energy than they expend or that they store the consumed fat more efficiently. However, when daily energy intake is compared in the cohort of subjects from NHANES III, men consumed more energy, even after adjusting for fat-free mass (187 kJkg^−1^ versus 170 kJkg^−1^) [1, 10]. One possible explanation is that women are more efficient at conserving energy and storing it as fat. Supporting this notion is the recognition that women must reduce their dietary intake by a greater proportion to achieve the same degree of weight loss as men [12]. Another observation is that in the first half of pregnancy, women increase their FM without evident increases in energy intake or decreases in expenditure. This ability to increase FM without substantial increases in energy intake points to the existence of metabolic adaptations that may contribute to the gender difference in FM.

### 3.1. Exercise Metabolism

Differences in rates of glucose and fat oxidation during exercise do not seem to explain the gender difference in FM. Women preferentially burn a higher fat-to-glucose fuel mixture during exercise [13]. Despite this, women lose less fat than men when faced with a similar energy deficit [14–17]. This may be related to more efficient fat storage during non-exercising periods [18, 19], considering less than 5% of the day is spent exercising in most healthy people. The higher fat mass in women may allow them to preferentially use this energy source as a fuel while exercising, whereas during the non-exercising times, women are storing fat more efficiently compared with men.

### 3.2. Postprandial Metabolism

Since women do not consume more energy compared to men, yet preferentially oxidise fat during exercise, it seems logical to propose that their higher FM is due to increased fat storage during non-exercising periods. Indeed, women were found to revert to a state of reduced fatty acid oxidation immediately after exercise, which persists for hours [21]. In addition, postprandial free fatty acid release from adipose tissue was reported to be lower in women than men [22, 23]. Several cross-sectional studies comparing men and women demonstrated that men oxidised a greater percentages of ingested fat [24, 25]. Using radiotracers, these authors also showed that postprandial fatty acid uptake by upper body subcutaneous and lower body adipose tissues were higher in women than men. Since the amount of energy expended in postabsorptive and postprandial states is greater than during exercise, this will have a great bearing on overall fat storage and FM.

Oestrogen is believed to be partly responsible for this reduction in postprandial fatty acid oxidation. Prospective studies using oral oestrogen therapy reported reductions in postprandial fatty acid oxidation. One study found significant reductions in postprandial fatty acid oxidation associated with a small increase in FM [27]. Similar changes were found in growth-hormone-deficient women on oral oestrogen therapy [28]. Another study reported larger reductions in postprandial fatty acid oxidation with oral oestrogen compared with transdermal therapy associated with a significant increase in FM [29]. This route-dependent observation raises the possibility that oral oestrogen therapy exerts its influence on the liver during first-pass metabolism. Therefore, studies using exogenous oestrogens have demonstrated that efficient fat storage in women was mediated through reduced postprandial fatty acid oxidation most likely because of an oestrogenic influence on hepatic processing of dietary fats.

However, the metabolic effect of exogenous oestrogen treatment may differ from endogenous oestrogens for several reasons. Exogenous synthetic oestrogens are generally more potent [30], and they have different pharmacokinetic attributes [31]. Several types of endogenous oestrogens exist, and each may have slightly different or synergistic actions [30]. Prospective studies during the hyperoestrogenic state of pregnancy are ideal for investigating the effects of endogenous oestrogens on postprandial fatty acid oxidation. However, the logistical difficulties of studying pregnant women prepregnancy has meant that to date, there has been limited prospective studies, of sufficient sample size, on energy metabolism during pregnancy. Spaaij et al. [32] studied 27 women from pre-pregnancy to delivery. They found that postprandial fat oxidation did not differ from prepregnant values during the first 13 weeks and actually increased afterwards. In cross-sectional studies, Nagy and King [33] detected no difference in postprandial fatty acid oxidation between 6 nonpregnant and 10 pregnant subjects, while a larger study (*n* = 23) detected a significant reduction in fatty acid oxidation in the pregnant group [34]. However, due to significant intersubject variations, findings from cross-sectional studies should be interpreted with caution.

In conclusion, a reduction in postprandial fatty acid oxidation has been shown to promote FM gain. Exogenous oestrogen treatment appears to induce this reduction, possibly by suppressing hepatic processing of dietary fats during first-pass metabolism. However, due to the complex actions of endogenous oestrogens, the small number of studies, and inconsistencies in study design, the effects of endogenous oestrogens on metabolism requires further research.

## 4. Regulation of Metabolism and Body Composition by Sex Hormones

Women's higher proportion of FM and the increase in FM during the first half of pregnancy may be due to the influence of sex hormones on metabolic processes such as lipolysis and fatty acid storage. In the literature reviewed, there is evidence to suggest that these effects can be mediated through hepatic targets, adipocyte targets, and adipokines such as leptin. However, how these pathways interplay is complex and generally poorly understood.

### 4.1. Hepatic Targets

Oestrogen may have an inhibitory effect on fatty acid oxidation on the liver, a major site on fatty acid metabolism. Several *in vitro* studies in murine hepatocytes showed that pharmacological concentrations of oestrogen reduced ketogenesis (a product of fatty acid oxidation) and increased fatty acid incorporation into triglycerides [9, 35]. Similar findings were reported in human subjects, where oral oestrogen therapy administered to hypogonadal and postmenopausal women reduced postprandial fatty oxidation and increased triglyceride levels [29, 36–38]. This indicates that exogenous oestrogen directs intrahepatic fatty acids away from oxidative pathways and into lipogenic pathways. 

In contrast, the effects of endogenous oestrogens are much harder to elucidate. Studies comparing women in follicular and luteal phases of the menstrual cycle detected no difference in energy metabolism, possibly because the change in oestrogen levels varies and there is also the influence of progesterone [24, 39]. Studies correlating oestrogen concentration and postprandial fatty acid oxidation in pregnant and non-pregnant subjects have looked at whole body metabolism of fat rather than isolating the effects on the liver. In addition, progesterone, which has been shown to have a synergistic and antagonistic effect, depending on the organ system, with oestrogen has not been studied in this context [8].

### 4.2. Adipocyte Targets

Oestrogen, progesterone and androgen receptors are present in adipose tissues [26]. As Table 2 shows, the expression of these receptors varies by depot and gender [40, 41]. Oestrogen receptors are higher in subcutaneous deposits in women, which may explain why women have greater subcutaneous gluteal and femoral deposits of fat [40, 41]. Genetic males with androgen insensitivity have a female body habitus [42], while women given exogenous androgens or suffering from virilising disorders will develop a male body habitus [40, 43–45]. Postmenopausal women experience an increase in waist to hip ratio and the amount of the visceral adipose tissue depot [32, 46, 47], which is partially reversed by oestrogen administration [48]. All this evidence indicates that the binding of sex hormones to their adipose tissue receptorspossibly promotes adipogenesis in some regions of the body. Although, it is known that many genes inadipocytes are transcriptionally regulated by sex hormones [26], the precise cellular mechanisms have not been fully elucidated.

### 4.3. Leptin

Leptin is an adipose tissue-derived hormone that inhibits fat gain by promoting hypophagia and hypermetabolism [49]. Thus, leptin has an important role in helping FM to remain relatively constant during adulthood. There is a gender difference in leptin levels which develops at puberty and is believed to be induced by sex hormones.

Leptin concentrations are higher per kilogram body weight in women than men. This difference is eliminated after adjusting for circulating concentrations of sex hormones [50]. Studies have found that leptin production was inhibited by androgens and promoted by oestrogens [26, 50]. Oestrogen has direct effects on FM as it upregulates leptin expression in adipocytes [51]. Central effects may also be present as oestrogen receptors have been detected in the hypothalamic nuclei controlling energy homeostasis. Circulating oestrogens are proposed to bind to these receptors and alter hypothalamic sensitivity to leptin-mediated signals, thus influencing secretion of leptin and possibly influencing metabolism and even fertility [52–54]. 

However, the relationship between leptin, oestrogen, and body composition is complex, as there is no change in leptin with menopause or with oestrogen replacement therapy [26]. Weight loss is associated with reduced leptin levels and hypogonadism [55]. In addition, the hyperandrogenism and diminished oestrogen surge in polycystic ovaries disease do not affect leptin levels [56, 57]. Therefore, the role of leptin in regulating FM is potentially influenced by oestrogen however, the mechanism of action is not completely clear.

## 5. Energy Balance in Pregnancy

Female reproduction requires increased amounts of energy. Yet, throughout history, women have carried their conceptus to term under a wide range of nutritional conditions. This suggests the presence of powerful metabolic adaptations [3]. During gestation, energy is required to grow the tissues of conception and reproduction, to maintain these tissues, and to prepare for lactation. Butte and King [58] found that an average weight gain of 13.8 kg, which includes 4.3 kg of fat, represents gestational energy needs. Based on this model, the Food and Agriculture Organization, World Health Organization, and United Nations University have calculated the energy requirement of pregnancy to be 360–370 MJ. This equates to an extra 1300 kJ/day, which is 15% above non-pregnant needs. However, gestational requirements have been shown to range from 30 MJ to 520 MJ in undernourished to overnourished women [59]. This variability points to the presence of metabolic adaptations for sustaining pregnancy under different nutritional conditions. 

In theory, this additional demand can be met by either increasing energy intake, decreasing expenditure, and/or mobilising fat stores. In contrast to expectations, numerous prospective and cross-sectional studies found that the first half of pregnancy is associated with little or no increase in energy intake [6, 60–62]. One study followed women prospectively through pregnancy and reported that energy intake in the first trimester of pregnancy is identical to pre-pregnancy [63]. Instead of reducing energy expenditure increased progressively during gestation [4, 64, 65]. Similarly, changes in diet-induced thermogenesis during pregnancy have not been consistently reported and therefore a reduction in diet-induced thermogenesis may not account for the positive energy balance [4, 33, 66–70]. 

These above observations raise the possibility that the energy costs of pregnancy are met by reductions in total energy expenditure. However, conclusive evidence fails to show that significant increases in energy intake or decreases in energy expenditure are the major contributors to the increase in FM in the first half of pregnancy. It is also important to note that pregnancy is a very plastic metabolic state, because even undernourished women can maintain FM [59]. Therefore, the cause of gestational fat gain may be mainly due to changes in metabolic pathways regulating the oxidation or storage of specific fuels especially fat.

## 6. Conclusion

Throughout their reproductive life, women maintain a higher proportion of body fat compared to men, and this difference is accentuated during the hyperoestrogenic state of pregnancy. However, studies have failed to demonstrate an energy surplus on all accounts. It is possible that women underestimate their food intake; however, some studies have reported that men underestimate their food intake compared with women [71]. The differences in physical activity between the sexes also need to be considered. Women do have a greater percent body fat, and it is possible that ovarian hormones, particularly oestrogen, may account for these observations by promoting postprandial conversion of dietary energy into fat. This theory needs to be supported by larger prospective studies and studies during natural hyper-oestrogenic states such as pregnancy. Oestrogens' actions may be mediated through hepatocyte and adipocyte targets and through regulation of hormones such as leptin. Further studies are needed to elucidate how these hormonal pathways interact and influence their targets. 

When considering lifestyle modifications, the sex difference in energy metabolism needs to be considered. Goals that take into account gender rather than just body weight or energy intake need to be utilised. Considering the high prevalence of obesity in modern society, it is important to understand the factors that regulate energy homeostasis and subsequently contribute to excess body fat. In the future, this understanding may culminate in strategies to control or reverse fat gain that do not only emphasise energy restriction.

## Figures and Tables

**Table 1:** Percentage FM in healthy non-Hispanic white men and women. Adapted from NHANES III [10].

Age range (years)	Males (%)	Females (%)	Difference (%)
12–13.9	18.4	24.8	6.4
20–29.9	21.8	31.0	8.2
30–39.9	23.6	33.0	9.4
40–49.9	24.2	35.4	11.2
50–59.9	25.1	37.3	12.2
60–69.9	26.2	36.9	10.7
70–79.9	25.1	35.9	10.8

**Table 2:** Expression of sex hormone receptors in adipose tissues. Adapted from Mayes, 2004 [26].

Receptor	Visceral fat		Subcutaneous fat	
	Female	Male	Female	Male
OR-*α*	+	+	++	+
OR-*β*	+	+	+++	+++
PR-A	−	−	+	−
PR-B	+	−	++	−
AR	++	++	+	+

OR oestrogen receptor; PR progesterone receptor; AR androgen receptor.
